# Supplementary material for: Short-term effects of national-level natural resource rents on life expectancy: A cross-country panel data analysis
Source: PLoS One. 2021 May 28;16(5):e0252336. doi: 10.1371/journal.pone.0252336 (PMC8162665; doi:10.1371/journal.pone.0252336)
Supplement: S1 Appendix — (DOCX) [file pone.0252336.s001.docx]

### S1 Appendix: Indicator definitions

| **Name** | **Definition** | **Source** |
| --- | --- | --- |
| GDP | GDP per capita is gross domestic product divided by midyear population. GDP is the sum of gross value added by all resident producers in the economy plus any product taxes and minus any subsidies not included in the value of the products. It is calculated without making deductions for depreciation of fabricated assets or for depletion and degradation of natural resources. Data are in constant 2010 USD. | World Bank national accounts data, and OECD National Accounts data files. |
| Total natural resources rents (% of GDP) | Total natural resources rents are the sum of oil rents, natural gas rents, coal rents (hard and soft), mineral rents, and forest rents. | The Changing Wealth of Nations: Measuring Sustainable Development in the New Millennium (World Bank, 2011). |
| Coal rents (% of GDP) | Coal rents are the difference between the value of both hard and soft coal production at world prices and their total costs of production. |  |
| Forest rents (% of GDP) | Forest rents are roundwood harvest times the product of average prices and a region-specific rental rate. |  |
| Mineral rents (% of GDP) | Mineral rents are the difference between the value of production for a stock of minerals at world prices and their total costs of production. Minerals included in the calculation are tin, gold, lead, zinc, iron, copper, nickel, silver, bauxite, and phosphate. |  |
| Natural gas rents (% of GDP) | Natural gas rents are the difference between the value of natural gas production at world prices and total costs of production. |  |
| Oil rents (% of GDP) | Oil rents are the difference between the value of crude oil production at world prices and total costs of production. |  |
| Population | Total population is based on the de facto definition of population, which counts all residents regardless of legal status or citizenship. The values shown are midyear estimates. | (1) United Nations Population Division. World Population Prospects: 2019 Revision. (2) Census reports and other statistical publications from national statistical offices, (3) Eurostat: Demographic Statistics, (4) United Nations Statistical Division. Population and Vital Statistics Reprot (various years), (5) U.S. Census Bureau: International Database, and (6) Secretariat of the Pacific Community: Statistics and Demography Programme. |
| Life expectancy at birth, total (years) | Life expectancy at birth indicates the number of years a newborn infant would live if prevailing patterns of mortality at the time of its birth were to stay the same throughout its life.  Life expectancy at birth used here is the average number of years a newborn is expected to live if mortality patterns at the time of its birth remain constant in the future. It reflects the overall mortality level of a population, and summarizes the mortality pattern that prevails across all age groups in a given year. It is calculated in a period life table which provides a snapshot of a population's mortality pattern at a given time. It therefore does not reflect the mortality pattern that a person actually experiences during his/her life, which can be calculated in a cohort life table.  High mortality in young age groups significantly lowers the life expectancy at birth. But if a person survives his/her childhood of high mortality, he/she may live much longer. For example, in a population with a life expectancy at birth of 50, there may be few people dying at age 50. The life expectancy at birth may be low due to the high childhood mortality so that once a person survives his/her childhood, he/she may live much longer than 50 years. | (1) United Nations Population Division. World Population Prospects: 2019 Revision. (2) Census reports and other statistical publications from national statistical offices, (3) Eurostat: Demographic Statistics, (4) United Nations Statistical Division. Population and Vital Statistics Reprot (various years), (5) U.S. Census Bureau: International Database, and (6) Secretariat of the Pacific Community: Statistics and Demography Programme. |
| Revenue, excluding grants (% of GDP) | Revenue is cash receipts from taxes, social contributions, and other revenues such as fines, fees, rent, and income from property or sales. Grants are also considered as revenue but are excluded here. | International Monetary Fund, Government Finance Statistics Yearbook and data files, and World Bank and OECD GDP estimates. |
| Control of Corruption: Estimate | Control of Corruption captures perceptions of the extent to which public power is exercised for private gain, including both petty and grand forms of corruption, as well as "capture" of the state by elites and private interests. Estimate gives the country's score on the aggregate indicator, in units of a standard normal distribution, i.e. ranging from approximately -2.5 to 2.5. | Kaufmann, Daniel, Aart Kraay and Massimo Mastruzzi (2010). "The Worldwide Governance Indicators: Methodology and Analytical Issues". World Bank Policy Research Working Paper No. 5430 (<http://papers.ssrn.com/sol3/>  papers.cfm?abstract_id=1682130). |
| Government Effectiveness: Estimate | Government Effectiveness captures perceptions of the quality of public services, the quality of the civil service and the degree of its independence from political pressures, the quality of policy formulation and implementation, and the credibility of the government's commitment to such policies. Estimate gives the country's score on the aggregate indicator, in units of a standard normal distribution, i.e. ranging from approximately -2.5 to 2.5. |  |
| Rule of Law: Estimate | Rule of Law captures perceptions of the extent to which agents have confidence in and abide by the rules of society, and in particular the quality of contract enforcement, property rights, the police, and the courts, as well as the likelihood of crime and violence. Estimate gives the country's score on the aggregate indicator, in units of a standard normal distribution, i.e. ranging from approximately -2.5 to 2.5. |  |
| Foreign Direct Investment, net inflow (% of GDP) | Foreign direct investment are the net inflows of investment to acquire a lasting management interest (10 percent or more of voting stock) in an enterprise operating in an economy other than that of the investor. It is the sum of equity capital, reinvestment of earnings, other long-term capital, and short-term capital as shown in the balance of payments. This series shows net inflows (new investment inflows less disinvestment) in the reporting economy from foreign investors, and is divided by GDP. | International Monetary Fund, International Financial Statistics and Balance of Payments databases, World Bank, International Debt Statistics, and World Bank and OECD GDP estimates. |
| Current health expenditure (% of GDP) | Level of current health expenditure expressed as a percentage of GDP. Estimates of current health expenditures include healthcare goods and services consumed during each year. | World Health Organization Global Health Expenditure database (http://apps.who.int/nha/database). |
| Capital health expenditure (% of GDP) | Level of capital investments on health expressed as a percentage of GDP. Capital health investments include health infrastructure (buildings, machinery, IT) and stocks of vaccines for emergency or outbreaks. | World Health Organization Global Health Expenditure database (http://apps.who.int/nha/database). |
| School enrollment,  tertiary (% gross) | Gross enrollment ratio is the ratio of total enrollment, regardless of age, to the population of the age group that officially corresponds to the level of education shown. Tertiary education, whether or not to an advanced research qualification, normally requires, as a minimum condition of admission, the successful completion of education at the secondary level. | UNESCO Institute for Statistics (http://uis.unesco.org/). |
| Prevalence of HIV | Prevalence of HIV refers to the percentage of people ages 15-49 who are infected with HIV. | UNAIDS estimates. |
| School enrollment,  secondary, female (% gross) | Gross enrollment ratio is the ratio of total enrollment, regardless of age, to the population of the age group that officially corresponds to the level of education shown. | United Nations Educational, Scientific, and Cultural Organization (UNESCO) Institute for Statistics. |
| Urban population | Urban population (% of total population) refers to people living in urban areas as defined by national statistical offices. | United Nations Population Division. World Urbanization Prospects: 2018 Revision. |
| energy index | Energy index contains 4.7% coal, 84.6% curde oil and 10.8% natural gas | World Bank Development Prospects Group. |
| precious metal index | Precious metals contain 77.8% gold, 18.9% silver and 3.3 platinum. |  |
