# Supplementary material for: Short-term effects of national-level natural resource rents on life expectancy: A cross-country panel data analysis
Source: PLoS One. 2021 May 28;16(5):e0252336. doi: 10.1371/journal.pone.0252336 (PMC8162665; doi:10.1371/journal.pone.0252336)
Supplement: S2 Appendix — (DOCX) [file pone.0252336.s002.docx]

### S2 Appendix: Correlation table

Table C1: Short run correlation matrix (multiple observation per country)

| Variables | (1) | (2) | (3) | (4) | (5) | (6) | (7) | (8) | (9) | (10) | (11) | (12) | (13) | (14) | (15) | |
| --- | --- | --- | --- | --- | --- | --- | --- | --- | --- | --- | --- | --- | --- | --- | --- | --- |
| (1) LE | 1 |  |  |  |  |  |  |  |  |  |  |  |  |  |  | |
| (2) GDP/Cap | 0.57 | 1 |  |  |  |  |  |  |  |  |  |  |  |  |  | |
| (3) Population | 0.04 | -0.05 | 1 |  |  |  |  |  |  |  |  |  |  |  |  | |
| (4) *Total Rents | -0.22 | -0.08 | -0.03 | 1 |  |  |  |  |  |  |  |  |  |  |  | |
| (5) *Revenue | 0.33 | 0.35 | -0.20 | 0.11 | 1 |  |  |  |  |  |  |  |  |  |  | |
| (6) Foreign Direct Investment | 0.11 | 0.08 | -0.04 | -0.01 | 0.18 | 1 |  |  |  |  |  |  |  |  |  | |
| (7) % of Urban Population | 0.71 | 0.60 | -0.06 | -0.04 | 0.36 | 0.09 | 1 |  |  |  |  |  |  |  |  | |
| (8) Prevalence of HIV | -0.49 | -0.17 | -0.07 | 0.04 | 0.04 | -0.02 | -0.28 | 1 |  |  |  |  |  |  |  | |
| (9) School enrollment, sec female | 0.86 | 0.55 | -0.04 | -0.25 | 0.40 | 0.11 | 0.68 | -0.24 | 1 |  |  |  |  |  |  | |
| (10) School enrollment, tertiary | 0.68 | 0.51 | -0.02 | -0.25 | 0.26 | 0.09 | 0.60 | -0.30 | 0.76 | 1 |  |  |  |  |  | |
| (11) *Current Health Expenditure | 0.27 | 0.15 | -0.08 | -0.38 | 0.16 | 0.07 | 0.20 | 0.04 | 0.31 | 0.49 | 1 |  |  |  |  | |
| (12) *Capital Health Expenditure | 0.06 | -0.02 | 0.00 | -0.09 | 0.06 | -0.03 | -0.04 | -0.04 | 0.05 | 0.10 | 0.12 | 1 |  |  |  | |
| (13) Rule of Law | 0.67 | 0.68 | -0.04 | -0.41 | 0.37 | 0.12 | 0.49 | -0.11 | 0.66 | 0.57 | 0.34 | 0.04 | 1 |  |  | |
| (14) Government Effectiveness | 0.71 | 0.77 | 0.01 | -0.39 | 0.34 | 0.10 | 0.53 | -0.14 | 0.70 | 0.61 | 0.27 | -0.01 | 0.93 | 1 |  | |
| (15) Control for Corruption | 0.64 | 0.77 | -0.06 | -0.37 | 0.34 | 0.11 | 0.50 | -0.08 | 0.64 | 0.54 | 0.33 | 0.02 | 0.94 | 0.93 | 1 | |
| Notes: * Measured as % GDP  Source: WDI | | | | | | | | | | | | | | | |  |
