# Supplementary material for: Short-term effects of national-level natural resource rents on life expectancy: A cross-country panel data analysis
Source: PLoS One. 2021 May 28;16(5):e0252336. doi: 10.1371/journal.pone.0252336 (PMC8162665; doi:10.1371/journal.pone.0252336)
Supplement: Attachment — Submitted filename: PONE-D-21-08827_Response to Reviewers.docx [file pone.0252336.s003.docx]

**Point-by-point response, PONE-D-21-08827**

Reviewer #1

*1. Is the manuscript technically sound, and do the data support the conclusions?*

*🡪 Reviewer #1: Partly*

**Response:** We thank Reviewer #1 for having scrutinized our manuscript and are grateful for the recommendations received. We hope that the reviewer is now fully satisfied with the quality of the paper after having addressed the reviewer’s concerns as specified below.

*2. Has the statistical analysis been performed appropriately and rigorously?*

*🡪 Reviewer #1: Yes*

**Response:** Thank you.

*3. Have the authors made all data underlying the findings in their manuscript fully available?*

*🡪 Reviewer #1: Yes*

**Response:** Thank you.

*4. Is the manuscript presented in an intelligible fashion and written in standard English?*

*🡪 Reviewer #1: Yes*

**Response:** Thank you.

*5. Review Comments to the Author:*

*The topic of the paper is unique, the level of methodology and evaluation is high, it is a very promising paper, however:
- introduction is very short and not appropriate regarding the content,
- there is absolutely no literature review in the paper,
- context isn't highlighted well,
- very short article as a whole.*

**Response:** We are pleased about the overall very positive appraisal of our study. In response to the reviewer’s request to provide more background while better embedding the paper in existing literature on the topic, we have now expanded the introduction for providing more background (see **lines 31-37, 58-69, 79-82** in the revised manuscript), which also includes additional references. Even though we do not have a formal literature section, we summarize the main literature on economic growth and the resource curse in the first two paragraphs for the introduction, and then provide an overview of the health-focused literature in lines (**58-69**) of the revised manuscript. We would be happy to move this part of the introduction to a separate “Literature review” section if this is preferred by the Editor and reviewer.

Moreover, we have also expanded the conclusion chapter of the paper (see **lines** **298-302**).

We hope that these additions have extended our work to the level that is sufficient. The overall article length is now just below 4000 words, which seems well aligned with word limits of most general interest journals.

Reviewer #2

*1. Is the manuscript technically sound, and do the data support the conclusions?*

*🡪 Reviewer #2: Yes*

**Response:** We thank Reviewer #2 for having scrutinized our manuscript and are grateful for the recommendations received.

*2. Has the statistical analysis been performed appropriately and rigorously?*

*🡪 Reviewer #2: Yes*

**Response:** Thank you.

*3. Have the authors made all data underlying the findings in their manuscript fully available?*

*🡪 Reviewer #2: Yes*

**Response:** Thank you.

*4. Is the manuscript presented in an intelligible fashion and written in standard English?*

*🡪 Reviewer #2: Yes*

**Response:** Thank you.

*5. Review Comments to the Author:*

*The topic is extremely interesting and inspiring. I consider it a very good idea to study the relationship between natural resources and human health. I also consider the database and the method used to be suitable for the study. Although the range of data processed can be identified, summarizing the categories in a table would improve clarity.*

**Response:** We are grateful for the overall positive evaluation of our study by Reviewer #2. We are sorry that the prior formatting of Table 1 (which has been fixed now) did not allow to see all the detail of the data. The full table contains means, standard deviation, min and max values as well as the temporal range, and should thus give readers a clear sense of the data used. We would of course be delighted to add further information or statistics if there are specific suggestions or requests.

*What I find problematic is that neither the title nor the abstract show that the study applies to some African countries. This may even be good, but it’s worth clarifying when positioning the article.*

**Response:** Our manuscript does indeed have an additional layer of analysis focusing on the African continent. In order to be more consistent on this aspect, as requested by Reviewer #2, the introduction now includes a few sentences that clarify why the study presented is of particular relevance for the African continent (lines **31-37**) and in the abstract we added an Africa-specific finding (lines **23-24**). We also clarified at the end of the introduction that we look both at Africa and the rest of the developing world (lines **79-82**). But we prefer not to include “Africa” in the title as we primarily present a global analysis, with Africa as a secondary, supplementary layer of analysis.

*The conclusion needs to be completed.*

**Response:** We have further expanded the conclusion of the paper (lines **298-302**).

*Editing errors need to be corrected (Table 1 hangs from the page.)*

**Response:** We have once more read our manuscript sentence-by-sentence and made corrections where needed. Also the layout of Table 1 was corrected.

Journal Requirements:

*When submitting your revision, we need you to address these additional requirements.*

1. *Please ensure that your manuscript meets PLOS ONE's style requirements, including those for file naming. The PLOS ONE style templates can be found at*

[*https://journals.plos.org/plosone/s/file?id=wjVg/PLOSOne_formatting_sample_main_body.pdf*](https://journals.plos.org/plosone/s/file?id=wjVg/PLOSOne_formatting_sample_main_body.pdf) *and*

[*https://journals.plos.org/plosone/s/file?id=ba62/PLOSOne_formatting_sample_title_authors_affiliations.pdf*](https://journals.plos.org/plosone/s/file?id=ba62/PLOSOne_formatting_sample_title_authors_affiliations.pdf)

**Response:** We have carefully studied the PLOS ONE's style requirements and applied them when developing the manuscript.

2. Thank you for stating the following in the Acknowledgments Section of your manuscript:

The authors would like to thank the Swiss Programme for Research on Global Issues for Development (r4d Programme), which is a joint funding initiative by the Swiss Agency for Development and Cooperation (SDC) and the Swiss National Science Foundation (SNSF) [grantnumber 169461] for having financed this study.

We note that you have provided funding information that is not currently declared in your Funding Statement. However, funding information should not appear in the Acknowledgments section or other areas of your manuscript. We will only publish funding information present in the Funding Statement section of the online submission form.

Please remove any funding-related text from the manuscript and let us know how you would like to update your Funding Statement. Currently, your Funding Statement reads as follows:

The funders had no role in study design, data collection and analysis, decision to publish, or preparation of the manuscript

Please include your amended statements within your cover letter; we will change the online submission form on your behalf.

**Response:** Many thanks for the clarification of the difference between the Funding Statement and the acknowledgements with PLOS ONE.

We have replaced the previous content of the acknowledgement section: “The authors would like to thank the entire team of the Health Impact Assessment for Sustainable Development (HIA4SD) Project both at the Swiss Tropical and Public Health Institute and partner institutions in the project countries for their continuous support over the course of this study” (lines **308-310**).

Kindly include the following content in the Funding Statement: “The authors would like to acknowledge the Swiss Programme for Research on Global Issues for Development (r4d Programme), which is a joint funding initiative by the Swiss Agency for Development and Cooperation (SDC) and the Swiss National Science Foundation (SNSF). The funders had no role in study design, data collection and analysis, decision to publish, or preparation of the manuscript.”
